# Mechanisms governing the impact of nitrogen stress on the formation of secondary metabolites in *Artemisia argyi* leaves

**DOI:** 10.1038/s41598-023-40098-5

**Published:** 2023-08-08

**Authors:** Zixin Wang, Tingting Zhao, Lin Ma, Changjie Chen, Yuhuan Miao, Lanping Guo, Dahui Liu

**Affiliations:** 1 Key Laboratory of Traditional Chinese Medicine Resources and Chemistry of Hubei Province, Hubei University of Chinese Medicine, Wuhan, 430065 China; 2 China Academy of Chinese Medical Sciences, Beijing, China

**Keywords:** Molecular biology, Plant sciences

## Abstract

Nitrogen is a key factor in various physiological and metabolic processes in plants. Providing an adequate supply of nitrogen is essential for improving the total yield and quality of the medicinal plant *Artemisia argyi* (*A. argyi*), but the underlying mechanisms of how this nutrient alters the crop remains unclear. In this study, we conducted a series of pot experiments to investigate the agronomic traits and active components in the leaves of *A. argyi* plants under low and high nitrogen stress. Additionally, we used transcriptome analysis and RT-qPCR to explore the molecular pathways associated with nitrogen stress. Our results demonstrate a dramatic increase in the accumulation of phenolic acids and flavonoids in the low nitrogen (LN) stress group compared to the control (CK), with increases of 40.00% and 79.49%, respectively. Interestingly, plants in the high nitrogen (HN) stress group exhibited enhanced plant growth with larger leaves, thicker stems, and a 3% increase in volatile oil content compared to the CK. Moreover, *A. argyi* in the HN group displayed a 66% increase in volatile oil concentration compared to the LN group. Our combined transcriptome and q-PCR results indicate that LN stress promotes the expression of genes involved in flavonoid synthesis, while HN stress promotes the expression of genes related to terpene skeleton production and photosynthesis. Taken together, these findings suggest that different gene expression levels under LN and HN stress contribute to the photosynthesis capacity and the accumulation of active ingredients in *A. argyi* leaves. Our results elucidate the physiological and molecular mechanisms of nitrogen stress on *A. argyi* secondary metabolites and guide fertilization strategies for plant cultivation.

## Introduction

Nitrogen is an essential nutrient for plant growth and development as it represents a major component of various proteins, nucleic acids, enzymes, chlorophyll, vitamins, and hormones^1^. The element plays a significant role in many aspects of a plant’s life, including the accumulation of secondary metabolites, growth and development, and productive yield. Its involvement in the synthesis of organic osmoregulatory substances and subsequent metabolism is crucial for enhancing plant physiology and agronomy^2^. For instance, a previous study demonstrated how nitrogen fertilizer application can significantly increase rice yield^3^.

Increasing soil nitrogen fertilization leads to a decrease in the non-structural carbohydrate content in plants, subsequently reducing the prevalence of monoterpenes that utilize these carbohydrates as substrates for synthesis. Modulating the level of nitrogen in the soil can be an effective method for altering plant chemistry and increasing the amount of volatile oils^4^. However, as soil nitrogen increases, so does the amount of secondary compounds that use amino acids as precursors^5^.

The distinct effects of nitrogen fertilizer on plant flavonoid content may be attributed to the carbon-nutrient balance hypothesis (CNB), which states that plant growth is more constrained under nitrogen stress than during photosynthesis, necessitating an increase in secondary metabolites for antioxidant accumulation^6^. Previous studies have demonstrated that excessive nitrogen leads to flavonoid decreases in *ginkgo*^7^, apple^8^, and buckwheat^9^. This decrease may be attributed to the reduced activity of the phenylalanine synthetic enzyme activity, which is crucial for the production of flavonoid secondary metabolites. In the presence of excess nitrogen, this enzyme exhibits diminished activity, thereby affecting the synthesis of free flavonoids^8,10^. Previous studies in medicinal plants have demonstrated that a reduction of this enzyme induced by high nitrogen levels leads to the inhibition of the synthesis of some phenolic acids like chlorogenic acid. Similarly, studies have shown that low nitrogen can increase the supply of total phenols in medicinal plants such as *Salvia miltiorrhiza Bunge*^11^, *Malaysian Kacip Fatimah*^12^, and nettle^13^, allowing for the increased formation of phenolic compounds.

*Artemisia argyi (Artemisia argyi* Levi. et Vant*)*, commonly known as Chinese mugwort or silvery wormwood, is an edible herb belonging to the *Asteraceae* family with traditional uses in medicine. The shrubby perennial is well known for its ability to stop bleeding, regulate menstrual flow, alleviate cold symptoms, and reduce pain. It contains a variety of volatile oils, flavonoids, polysaccharides, and many other valuable active ingredients. In traditional Chinese medicine, a practice known as moxibustion uses the burning of the plant to externally treat or prevent diseases. Extracts are also commonly made from the leaves of *A. argyi* for use as a supplement called wormwood. Both chemical and organic fertilizers can be used in *A.argyi* cultivation to induce thicker stems, larger leaves, and higher extract yields^14^. *A.argyi* leaves grown in Herb Spring, located in the Hubei Province of China, exhibit nearly double the yield of volatile oil compared to plants from other locations. This characteristic not only enhances their medicinal properties but also increases their economic value^15^*.*

As an essential nutrient for growth and development, nitrogen can be effectively used to increase the yield and quality of medicinal plants. The majority of nitrogen in terrestrial ecosystems exists as atmospheric nitrogen, or N_2_, a gas that cannot be directly absorbed and used by plants. Bioavailable nitrogen is scarce, leading to restrictions in plant growth and production, significantly influencing the structure and functions of ecosystems. To overcome the lack of naturally occurring nitrogen, modern growers frequently supplement their soils with fertilizers to maintain and improve crop yields^13^. However, excessive nitrogen can reduce plant disease resistance, hindering the capacity for the accumulation of active ingredients in medicinal plants. The excessive use of nitrogen fertilizer^16^ can also increase soil caking^17^ and other potentially serious environmental consequences. Hence, it is crucial to determine the dosage of nitrogen fertilizer that will maximize the quantity and quality of *A. argyi* leaves without contaminating the surrounding ecosystems.

In this study, we hypothesized that varying degrees of nitrogen stress affect agronomic traits and the contents of active ingredients in *A. argyi* leaves by regulating related genes and metabolic pathways. This study aims to provide a better understanding of the molecular effects of nitrogen on *A. argyi* from a genetic and metabolic perspective.

## Results

### Nitrogen fertilizer promotes the growth of A. argyi

**Figure 1:**
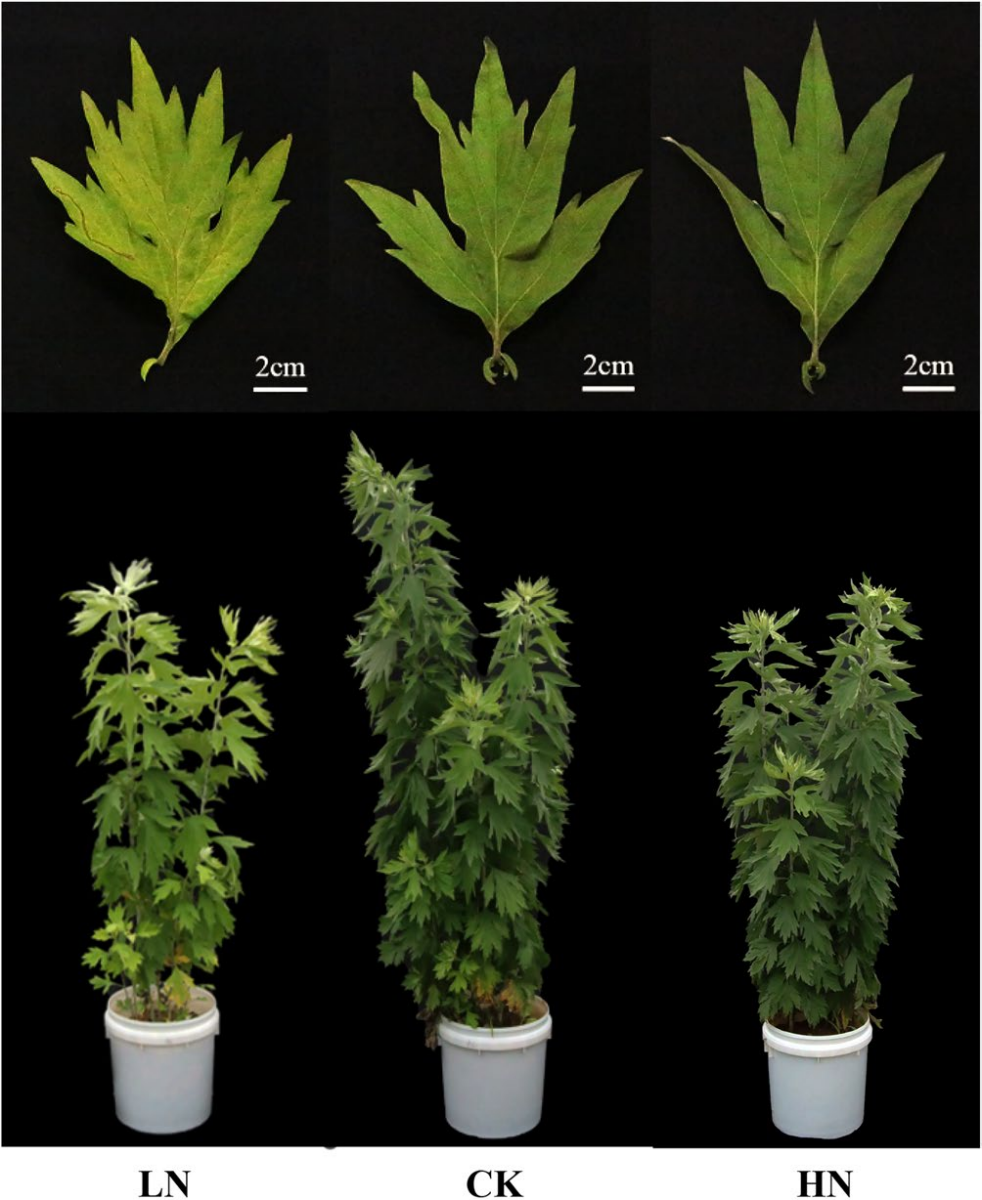
Morphological effects of low, control, and high nitrogen application rates on *A. argyi* leaves and plants.

**Figure 2:**
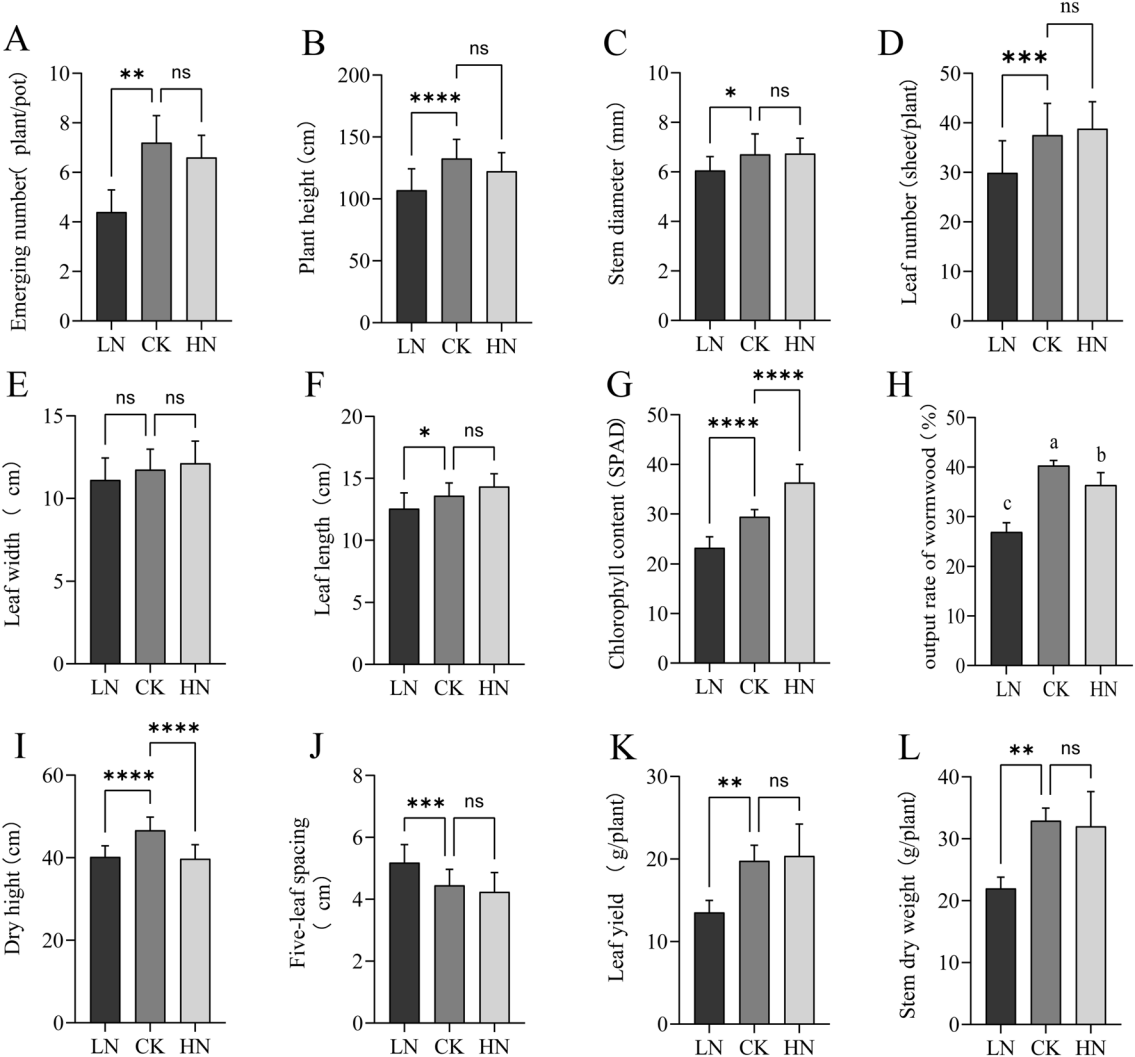
Effects of low, control, and high nitrogen application rates on important agronomic traits of *A. argyi.* Error bars represent the SD (n = 3). Statistical significance was determined via an LSD test at ^ns^p > 0.05, *p < 0.05, **p < 0.01, ***p < 0.001.

In this study, pot experiments were conducted to explore the effects of low (LN) and high (HN) nitrogen stress on agronomic traits and the accumulation of effective components in *A. argyi*. *A. argyi* plants were divided into three groups, each of which was treated with a different dosage of nitrogen fertilizer (LN 0.5 g/10 kg, CK 2 g/10 kg, HN 4 g/10 kg). Our results show that compared to the control group (CK), plants in the HN group displayed greener leaves, while plants in the LN group showed symptoms of nitrogen deficiency with yellow-green leaves and short stature (Fig. 1). We also found that both LN and HN treatment groups showed a decreased germination compared to the CK (Fig. 2A). Moreover, the CK group represented the largest plant height, dry plant height, dry stem height, and output of wormwood (Fig. 2B,I,K,H). The stem diameter, number of blades, leaf width, leaf length, and chlorophyll content were highest in the HN group (Fig. 2C,D,E,F,G), however, it showed a decrease in five-leaf spacing (Fig. 2J). Additionally, both the CK and HN groups showed significantly increased leaf weights, stem dry weights, and wormwood output rates compared to the LN group, with the highest output rate in the CK (Fig. 2J,K,L). Taken together, these results demonstrate that nitrogen fertilizer promotes the growth, development, and wormwood output rate of *A. argyi* leaves.

### Concentration of elements in A.argyi leaves under LN and HN stress

**Figure 3:**
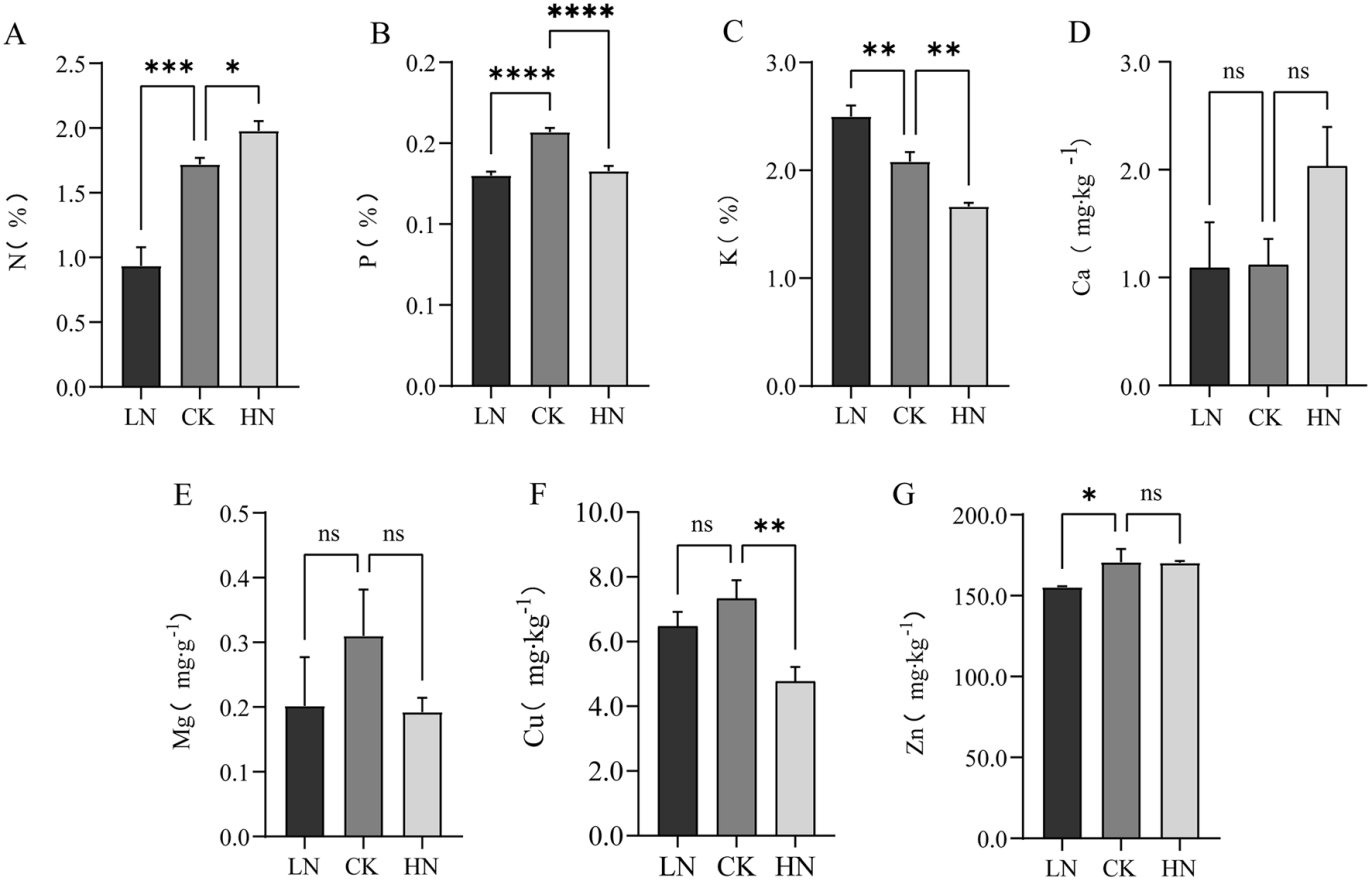
Effects of low, control, and high nitrogen application rates on the abundance of mineral elements in *A. argyi.*

Previous research has demonstrated that barley plants undergoing HN stress contain higher levels of other crucial elements (P, K, Ca, Fe, Cu, Mn)^18^. Here, we analyzed the quantities of various elements in *A. argyi* leaves treated with differing levels of nitrogen and found that the HN group contained the highest nitrogen (1.98%) but the lowest K content (1.67%); the CK group contained the highest P content (0.157%); and the LN group contained the highest K content (2.50%). Additionally, in the LN, CK, and HN groups, the Mg content was 0.17, 0.31, and 0.19 mg/g; the Cu content was 6.45, 7.33, and 4.77 mg/kg; and the Zn content was 155.19, 170.76, and 170.24 mg/kg, respectively. The N and Ca levels were highest in the HN group, whereas the P, Mg, Cu, and Zn levels peaked in the CK group. The K content decreased most dramatically under HN stress (Fig. 3).

### HN stress promotes the accumulation of volatile oil and its components in A. argyi

**Figure 4:**
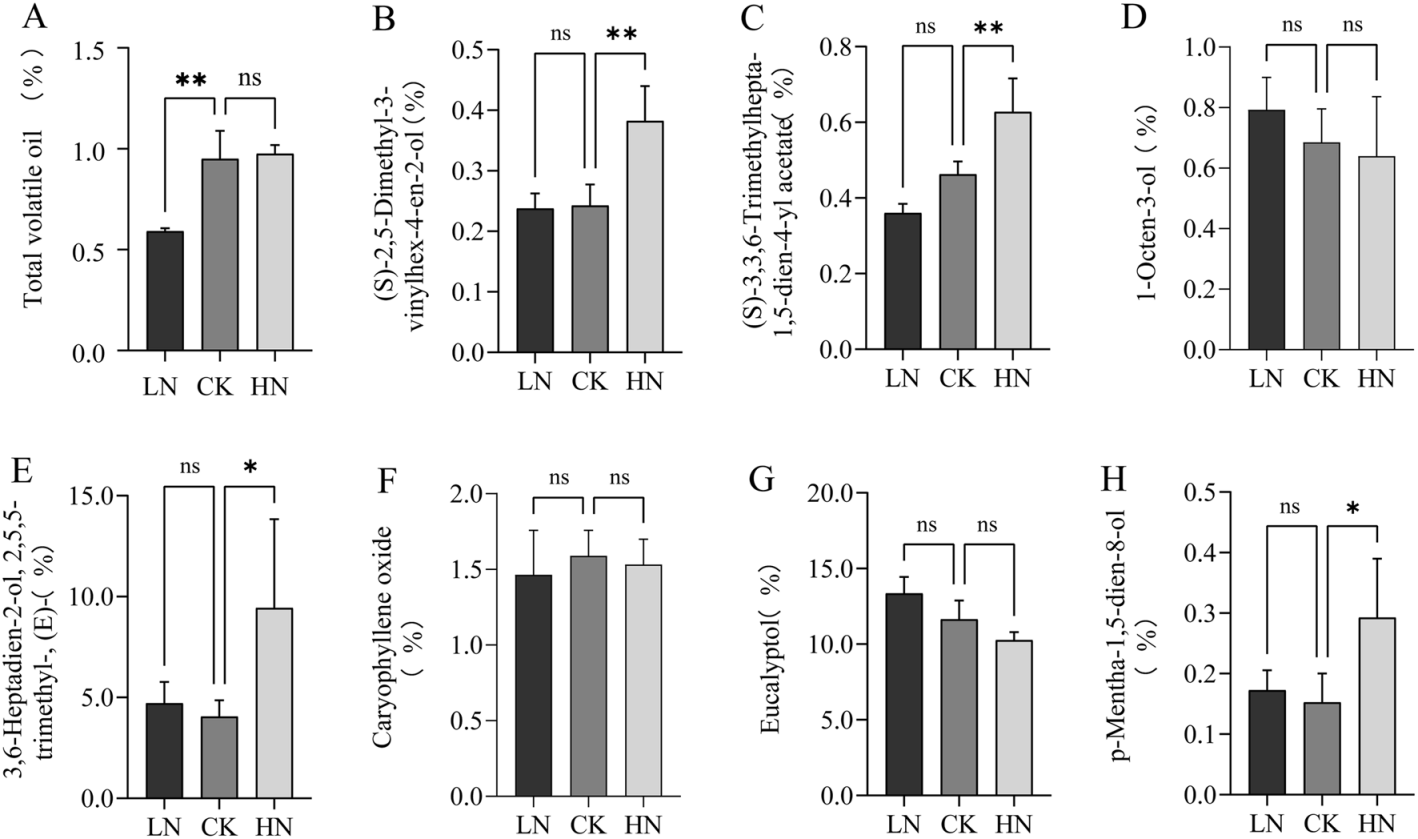
Effects of low, control, and high nitrogen application rates on the total quantity and composition of volatile oils in *A. argyi* leaves.

Volatile oils, flavonoids, and phenolic acids are important secondary metabolic components in *A. argyi*. To explore the effects of nitrogen application on the accumulation of volatile elements, we determined the total volatile oil and volatile components in *A. argyi* leaves under LN, CK, and HN treatments. Compared to the LN group, we observed a 60.56% and 64.79% increase in volatile oil content in the CK and HN groups, respectively. There were no significant differences in oil content between the CK and HN groups (Fig. 4A). A more detailed analysis of the volatile substances revealed a drop in the relative percentage of eucalyptol from 12.86% to 23.12% in the CK and HN groups compared to LN. There was no significant difference in the relative percentage contents of 3, 6-Heptadien-2-ol, 2, 5, 5-trimethyl-, (E)-, (S)-2, 5-dimethyl-3-vinyl hex-4-en-2-ol and p-Mentha-1, 5-dien-8-ol between the LN and CK groups, but there was a significant increase in the HN group. Similarly, the relative percentage of (S)-3, 3, 6-Trimethylhepta-1, 5-dien-4-yl acetate increased significantly with the rise in nitrogen fertilizer application, increasing from 28.47% to 74.31%. Finally, the relative percentage of 1-octen-3-ol and caryophyllene oxide showed no significant changes to the nitrogen treatments (Fig. 4B–H). These results suggest that HN stress promotes the production of total volatile oils, with various volatile components displaying their own distinct trends.

### LN stress promotes the accumulation of phenolic acids and flavonoids in A. argyi leaves

**Figure 5:**
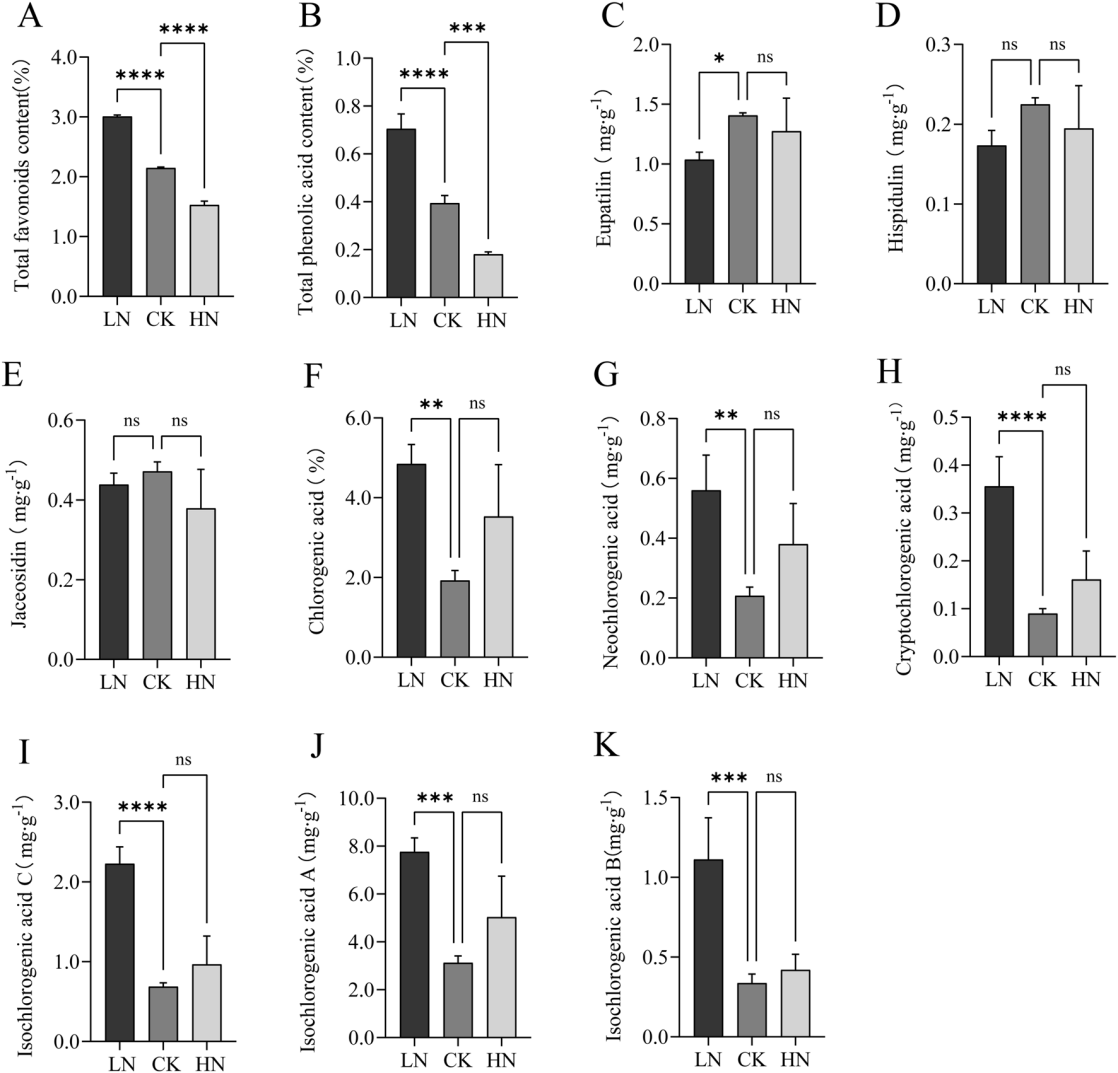
Effects of low, control, and high nitrogen application on the concentration of active ingredients in *A. argyi* leaves. Total flavonoid content (**A**) represents the combination of all measured flavonoids (**C**–**E**). Total phenolic acid content (**B**) represents the combination of all measured phenolic acids (**F**–**K**).

Next, we measured the quantities of flavonoids and phenolic acids in *A. argyi* leaves under differing levels of nitrogen stress. Our results demonstrate that the increase in nitrogen heavily impacted the abundance of flavonoids and phenolic acids in *A. argyi* plants, decreasing their amounts by 44.02%-74.39% and 28.57%-49.17%, respectively (Fig. 5A,B). Higher levels of nitrogen also led to an initial increase in the amount of eupatilin and hispidlin, but the elevated levels did not persist. Compared to the LN group, the content of jaceosidin rose by 6.82% and 35.58% in the CK and HN groups, respectively (Fig. 5C,D). There was no significant difference in the amount of jaceosidin between treatments (Fig. 5E). The contents of chlorogenic acid, neochlorogenic acid, cryptochlorogenic acid, isochlorogenic acid C, isochlorogenic acid A, and isochlorogenic acid B were all highest in the LN treatment (Fig. 5F–K) and lowest in the CK treatment. Together, these results demonstrate that LN stress promotes the accumulation of total phenolic acids and flavonoids.

### Correlation analysis between mineral elements, agronomic traits, and active ingredients of A. argyi leaves under nitrogen stress

**Figure 6:**
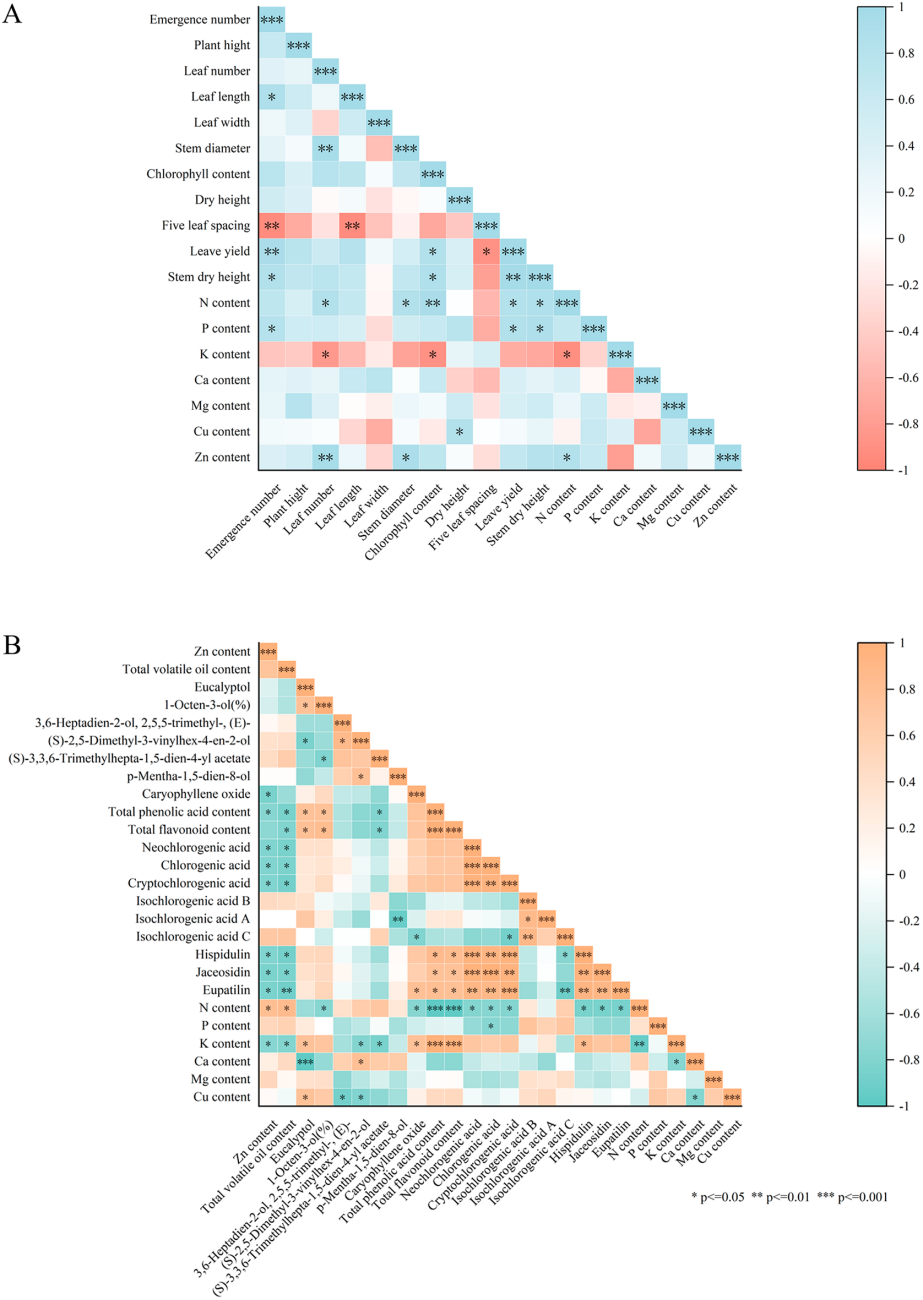
Correlation analysis between the levels of various mineral elements and the agronomic traits (**A**) and active ingredients (**B**) of *A. argyi* leaves under different nitrogen stress conditions.

During this study, we observed correlations in *A. argyi* leaves between seven mineral elements, agronomic traits, and the concentrations of various active ingredients (Fig. 6). In total, we identified 19 positively and 6 negatively correlated traits. We found that N content was positively correlated with leaf number, stem diameter, chlorophyll content, leaf yield, and stem dry weight; P was positively correlated with emerging number, leaf yield, and stalk dry weight; K was negatively correlated with leaf number and chlorophyll content; Cu was positively correlated with withering height; Zn was positively correlated with leaf number and stem thickness; N and K were negatively correlated; and N and Zn were positively correlated.

We found significant correlations (correlation coefficients > 0.01) between the active ingredients and the leaf components in 87 trait pairs, 43 of which were positively correlated while the remaining 44 were negatively correlated (Fig. 6). In addition to volatile oil content, N was found to be negatively correlated with total phenolic acid, total flavonoids, neochlorogenic acid, chlorogenic acid, and total cryptogenic acid; P was negatively correlated with neochlorogenic acid; and K was positively correlated with all these variables plus caryophyllene oxide and eucalyptol content. Additionally, K elements showed a positive relationship with volatile oil content, total phenolic acid, total flavonoids, caryophyllene oxide, and eucalyptol. Cu was positively correlated with eucalyptol content and negatively correlated with 3,6-Heptadien-2-ol, and egatively correlated with2,5,5-trimethyl-, (E)-, (S)-2,5-Dimethyl-3-vinylhex-4-en-2-ol content. Ca was significantly negatively correlated with eucalyptol content.

We also noted both antagonistic and synergistic interactions among the seven analyzed elements. Combined, these results elucidate the interaction between the uptake and transport of mineral elements in *A. argyi* leaves undergoing different N treatments.

### Functional classification and enrichment analysis of deferentially expressed genes in A. argyi under nitrogen stress

Next, we performed transcriptomic analyses on *A. argyi* leaves to investigate the molecular mechanisms by which nitrogen fertilizers affect plant growth and quality. We identified a total of 2,883 DEGs in leaves under LN stress compared to the CK group. These DEGs are comprised of 1,623 up-regulated genes and 1,260 down-regulated genes. In the HN group, we found that 265 genes were up-regulated and 282 genes were down-regulated compared to the CK group.

**Figure 7:**
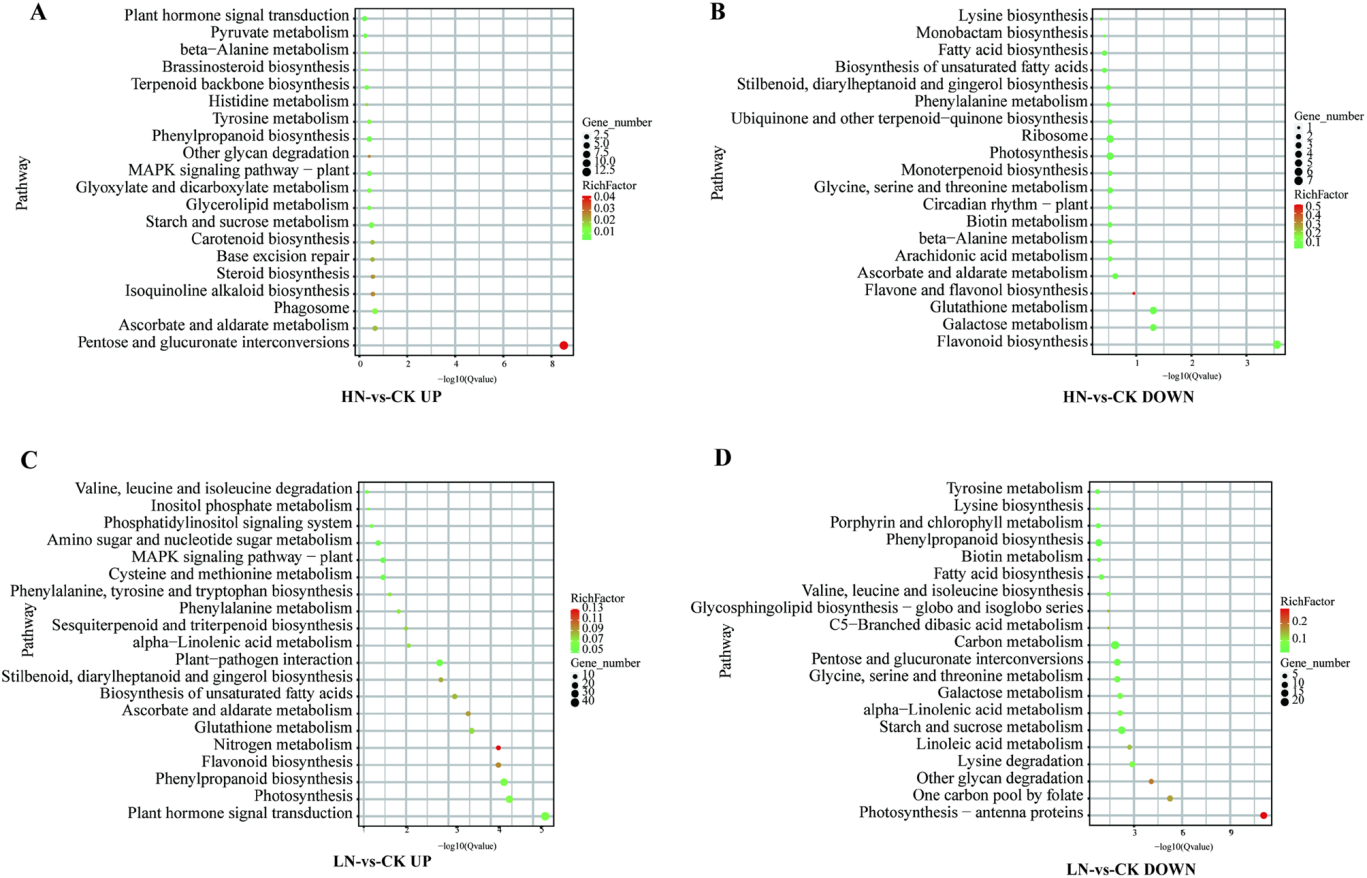
KEGG enrichment analysis of *A. argyi* leaves following low, control, and high applications of nitrogen. Functional enrichment of genes up-regulated (**A**) and down-regulated by HN-VS-CK. Functional enrichment of genes up-regulated (**C**) and down-regulated (**D**) by LN-VS-CK.

Because different stresses have a significant impact on the amount of secondary metabolites in Artemisia argyi leaves, we focused our functional localization on the production pathways of flavonoids, phenolic acids, and terpenoids in order to further characterize the effects of nitrogen stress on the synthesis pathways of major *A. argyi* secondary metabolites. We carried out functional and pathway analyses using the Kyoto Encyclopedia of Genes and Genomes (KEGG) (http://www.genome.jp/kaas-bin/kaas_main?mode=est_b;22), theseidentified genes were subjected to KEGG enrichment analysis^19–21^ (Fig. 7). We first investigated the DEGs under LN stress compared to the CK group. Of the up-regulated DEGs, 15 were enriched in "flavonoid biosynthesis", which involves enzymes such as chalcone synthase (CHS), chalcone isomerase (CHI), and flavanone 3-hydroxylase (F3H). An additional 30 genes were enriched in "phenylpropane biosynthesis", which involves enzymes such as phenylalanine ammonia-lyase (PAL), cinnamate 4-hydroxylase (C4H), and p-Coumaroyl-CoA ligase (4CL). Of the down-regulated DEGs, 15 were enriched in "phenylpropane biosynthesis". Subsequently, we analyzed the identified DEGs in the HN group compared to the CK group. Two of these genes were enriched in the "terpene skeleton biosynthesis" pathway, which involves the enzymes such as geranyl diphosphate synthase(GPPS), farnesyl diphosphate synthase (FPPS), geranylgeranyl diphosphate synthase (GGPPS), and terpene synthases (TPS). Four genes enriched in the "phenylpropane biosynthesis" pathway were up-regulated, while an additional seven were down-regulated in the same category (Fig. 7). These results demonstrate the effect of nitrogen stress on the biosynthesis of flavonoids and terpenes.

### Major genes involved in the metabolic pathways of terpenoids, flavonoids, and phenolic acids under LN and HN stress

**Figure 8:**
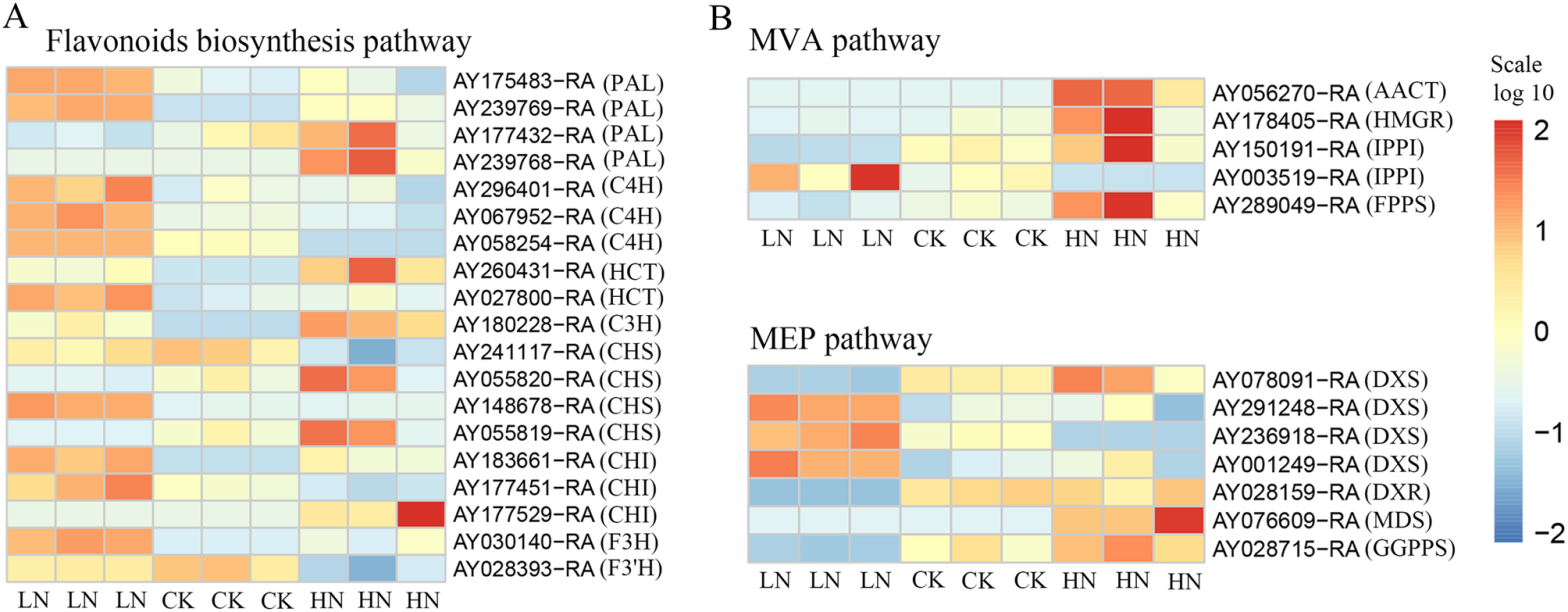
Heat map of major metabolic enzymatic pathways under low, control, and high nitrogen application. Genes involved in the flavonoid biosynthesis pathways (**A**) and the MVA and MEP synthesis pathways (**B**).

Flavonoids and terpenoids are the most important active ingredients in *A. argyi* leaves. In this study, we compared the gene expressions of these two compound classes in leaves subjected to nitrogen stress and visualized the results using a heat map (Fig. 8). In the flavonoid synthesis pathway, there was a significant decrease in the expressions of *AY175438-RA* and *AY239769-RA*, two genes that encode phenylalanine ammonia-lyase 1 (PAL) synthesis, in CK and HN groups compared to the LN group.

Conversely, *AY177432-RA, AY239769-RA, AY067952-RA,* and *AY058254-RA*, which encode for trans-cinnamate 4-monooxygenase (C4H) synthesis, showed the highest expressions under LN stress. Moreover, LN also displayed high expression levels of *AY183661-RA* and *AY1774451-RA*, which encode chalcone flavanone isomerase (CHI), as well as other genes that regulate enzymes required for flavonoid synthesis. The HN group showed higher expression levels of genes related to the mevalonate (MVA) pathway, which is associated with terpene skeleton synthesis (Fig. 8). In this treatment, there were elevated expression levels of *AY056270-RA* which encodes acetyl-CoA acetyltransferase (AACT) synthesis; *AY-178405-RA* which encodes 3-hydroxy-3-methylglutaryl coenzyme A 3-hydroxy-3-methylglutaryl coenzyme A reductase (HMGR); *AY-003519-RA* which encodes isopentenyl diphosphate isomerase (IPPI) synthesis; and *AY-289049-RA* which encodes farnesyl diphosphate synthetase (FPPS) synthesis. The MEP synthesis pathway was more mixed, with 1-deoxy-D-xylulose 5-phosphate synthase (DXS) encoding genes *AY291248-RA, AY236918-RA,* and *AY001249-RA* showing higher expression under LN stress, while *AY-078091-RA* and *AY-028159-RA* showed higher expression under HN stress. Finally, HN treatments were also elevated in the expression of *AY-076609-RA* which encodes 2-C-methyl-D-erythritol-2, 4-cycloid-phosphate synthase (MDS) synthesis; *AY-076609-RA* which encodes MDS synthesis; and *AY-028715-RA* which encodes geranyl diphosphate synthase (GGPPS) synthesis.

### RT-qPCR validation of DEGs

**Figure 9:**
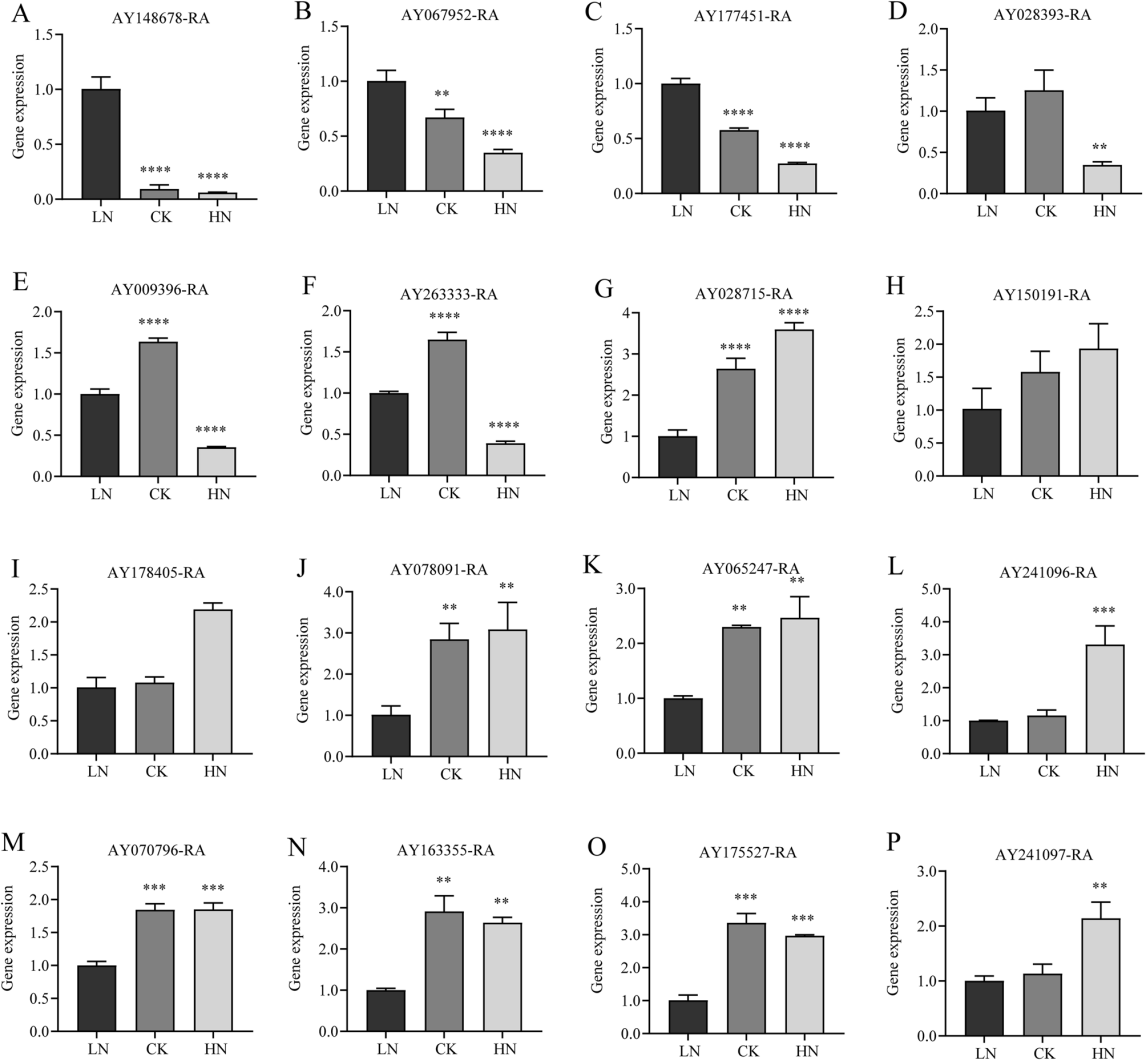
RT-qPCR analysis of relative expression levels of related genes in *A. argyi*. The values are presented as mean ± SD of three independent experiments employing randomly mixed samples. Different levels of significant variation are denoted by **, ***, and ****. ** $\le$ 0.05, *** $\le$ 0.01, and **** $\le$ 0.001.

Next, we verified key genes involved in the synthesis pathways of phenolic acids, flavonoids, and terpene synthesis in *A. argyi* leaves by RT-qPCR. We found that an increase in nitrogen application significantly decreased the expression levels of *AY148678-RA* (encoding CHS), *AY067952-RA* (encoding C4H), and *AY177451-RA* (encoding CHI) (Fig. 9A,B,C). Similarly, the expression levels of *AY028393-RA* (encoding F3'H), *AY009396-RA* (encoding CHS), and *AY2633333-RA* (encoding CHS) were the lowest under HN (Fig. 9,E,F). The expression of *AY028715-RA* (encoding GGPPS), *AY150191-RA* (encoding isopentenyl diphosphate isomerase-IPPI), *AY178405-RA* (encoding HMGR), and *AY078091-RA* (encoding DXS) however, were elevated in the HS group (Fig. 9G,H,I,J).

Additionally, we quantitatively confirmed the expression of several genes essential for photosynthesis. In the HN group, there was a distinct increase in the expression of key genes *AY065247-RA* and *AY241096-RA*, both of which are involved in the photosynthesis pathway. The expression of *AY065247-RA* was significantly down-regulated under LN stress and up-regulated under HN stress, while the expression of *AY241096-RA* showed no differences between LN and CK (Fig. 9K,L). *AY070796-RA*, *AY163355-RA*, *AY175527-RA*, and *AY241097-RA* are all involved in the chloroplast synthesis pathway, and their expression levels were significantly higher in the CK and HN groups compared to the LN group (Fig. 9M,N,O). The expression of *AY241097-RA*, however, was significantly up-regulated under HN stress (Fig. 9P). These results are consistent with our transcriptomic analyses.

## Discussion

Many previous studies have demonstrated the significant role N plays in sustaining plant development and metabolism. Fertilizers rich in nitrogen are often used by growers to increase crop yields, but their overuse tends to degrade plant quality and leads to secondary soil salinization and energy waste. Hence, it is crucial to determine the ideal amount of supplemental N used in growing operations to ensure a balance between plant quality and the environment.

Plants can create amino acids by directly combining the salts of ammonium nitrogen with organic acids created during photosynthetic reactions^22^, which in turn are capable of producing additional nitrogenous organic matter. *A. argyi* plants are small (Fig. 1) with feeble stalks (Fig. 2C) and yellow leaves (Fig. 1). Our findings reveal that the chlorophyll content (Fig. 2G) in *A. argyi* leaves significantly increases in higher nitrogen environments, which is consistent with previous research^23^.

*A.argyi* leaves are rich in volatile oils, many of which are extremely important in traditional Chinese medicine. Here, we determined the volatile oil content of *A. argyi* leaves undergoing various nitrogen treatments and found them to be the lowest in the LN group. There was no significant difference between the CK and HN groups, which may be explained by the effects of nitrogen on growth. Flavanoids are also incredibly important plant components, and previous research has proposed that select flavonoids have anti-inflammatory, anti-cancer, anti-hypercholesterolemic, and antioxidant properties^24,25^. To date, > 20 flavonoids, flavanols, and their glycosides^26–29^ have been isolated from *A. argyi* leaves. In this study, we found that the total flavonoid content in *A. argyi* leaves decreases with high levels of applied nitrogen. We observed that eupatilin, hispidulin, and jaceosidin were most prevalent in the CK group. We also measured phenolic acid content and found them to be significantly more present in the LN group than in the CK and HN groups. Next, we assessed the synergistic and antagonistic effects of mineral element content with plant growth species and active ingredient content using Pearson’s correlation coefficient. Our results showed that N content in *A. argyi* leaves was significantly positively correlated with photosynthesis and negatively correlated with flavonoid and phenolic acid content. This information provides a reference for future research in determining the effects of N application on metabolic pathways in *A. argyi* leaves. Our results support the CNB hypothesis which predicts that a higher level of carbon-based secondary metabolites, such as polyphenols, are present under limited nitrogen availability^30^. In turn, decreased nitrogen could have accelerated the metabolism of phenylpropanoids, increasing the concentration of total polyphenols in plant tissues. In this study, we identified and analyzed differentially expressed genes enriched under various nitrogen treatments to understand their functional impact. We found several enriched genes associated with the synthesis of phenylalanine, flavonoids, and terpenoid skeletons. Intermediates like PAL and C4H are among the precursors for flavonoid formation. Additionally, CHS/CHI catalyzes the formation of flavanones. Our study shows that an elevated supply of nitrogen decreases the expression of *AY-175438RA* and *AY239769-RA*, genes that control the synthesis of phenylalanine ammonia-lyase 1 (PAL). This contrasts with *AY177432-RA* and *AY239769-RA*. Furthermore, we noted that the gene regulating trans-cinnamate 4-monooxygenase (C4H) synthesis is most expressed under LN conditions. Our results show that under LN conditions, there are high expression levels of chalcone flavanone isomerase (CHI), which catalyzes the formation of flavanones, as well as other genes controlling enzymes in the flavonoid synthesis pathway like *AY-067952RA* and *AY-058254RA*.

Plant terpenoids are synthesized independently via two major pathways–the mevalonate (MVA) pathway in the cytoplasm, and the methylerythritol 4-phosphate (MEP) pathway in the plastid. HMGR is an enzyme heavily involved in the MVA pathway, which catalyzes the formation of MVA from HMG-CoA. It is considered to be the first rate-limiting enzyme regulating cytoplasmic terpenoid metabolism. The IPP is a common intermediate in both pathways and can cross the plasma membrane, creating an intersection between the two^31^. DXS is an important rate-limiting enzyme in the DXP pathway, which catalyzes the condensation of pyruvate and 3-phosphoglyceraldehyde to form DOXP, leading to the formation of carotenoids, plastoquinones, and chlorophylls^32^. In this study, we identified several genes including *AY56270-RA*, *AY003519-RA*, and *AY289049-RA*, which were highly expressed in the LN treatment and are associated with the MEP synthesis pathway. Genes related to DXS enzyme synthesis like *AY291248-RA, AY236918-RA*, and *AY001249-RA* also displayed higher expression levels under LN conditions, while genes with similar functions like *AY078091-RA*, and *AY028159-RA* were more highly expressed under HN treatments.

In this study, we employed transcriptomic analysis to investigate the regulation of flavonoids, phenolic acids, and terpenoid secondary metabolites in *A. argyi* leaves following the application of varying nitrogen dosages. We also evaluated the regulatory genes associated with the production of enzymes and compared their expression levels between nitrogen treatment groups. Further studies are needed to validate the functions of these related genes and secondary metabolites in *A. argyi* leaves. Additionally, our research provides guidance for methods to improve plant development, maximize production, and increase active components. We aim to provide growers with pertinent information so they may establish effective and environmentally nitrogen fertilization routines. Our findings also seed to guide sustainable agricultural practices and enhance resource efficiency, benefiting both the environment and the production of *A. argyi*.

## Methods

### Plant materials and growth conditions

The pot experiments were conducted from January to June 2021 at the Medicinal Plant Garden of Hubei University of Traditional Chinese Medicine (30° 27′ N, 114° 15′ E). Each pot was filled with 8 kg of soil with 2 kg sand added to improve ventilation. The soil was yellow–brown and contained the following basic physical and chemical properties: pH, 5.92; organic matter, 2.6 g kg^–1^; total nitrogen, 0.4 g kg^–1^; available nitrogen, 21.4 mg kg^–1^; available phosphorus, 2.6 mg kg^–1^; and available potassium, 60.7 mg kg^–1^. Three herbaceous *A. argyi* rhizomes obtained from Qichun County in Hubei Province of China were planted in each pot in January 2021 at a depth of 3 cm. The quantities of nitrogen in each treatment were based on the Ma et al. nitrogen fertilization field trial of *A. argyi* in Qichun, Hubei^33^. The treatments contained 0.5 (LN), 2 (CK), and 4 (HN) g∙10 kg^−1^ of nitrogen and were applied three times in total. The first application was the largest and consisted of 60% of the total fertilizer applied in January 2021. The remaining 40% was divided into two separate applications on March 23 and April 11. Additionally, we applied 1.5 g∙10 kg^−1^ of P_2_O_5_ and 2 g∙10 kg^−1^ of K_2_O to each treatment and added 10 mL of an Arnon trace element mixture (Ca(NO_3_)_2_ 945 mg/L, K_2_SO_4_ 607 mg/L, NH_4_H_2_PO_4_ 115 mg/L, and Mg_2_SO_4_ 493 mg/L) to each basin to supplement trace nutrients. After determining the emergence rate, the plants were thinned to four plants per pot and routinely managed. All methods were performed in accordance with the relevant guidelines and regulations.

### Investigation of agronomic traits and yield

The emergence rate of *A. argyi* plants was investigated on March 2, 2021. Agronomic traits including plant height, stem diameter, dead leaf height, leaf number, five-leaf spacing, leaf width, leaf length, and chlorophyll content were measured on June 8, 2021. The chlorophyll content was measured with a chlorophyll meter (SPAD-502PLUS; Minolta, Tokyo, Japan). The plants were then harvested and dried in the shade. The total dry weight of *A. argyi* leaves and stems in each pot was measured and divided to calculate the weight per plant.

### The extraction of A. argyi wormwood

A total of 10 g of dry leaves from every each pot were used for the wormwood extraction. Each sample was placed on a high-speed universal pulverizer (OAK, China) with a rotating speed of 28,000 r·min^−1^ for 30 s. The crushed mixture was then placed into a 2 mm sieve to remove the powder, leaving only the wormwood extract. The wormwood output rate was calculated as the ratio of wormwood to dry leaf.

### Determination of mineral element content

The leaves of *A. argyi* plants grown under each nitrogen treatment were harvested, dried in the shade, and crushed. The quantity of P was measured using the vanadium molybdate blue colorimetric method, and K, Mg, Ca, and Zn were measured using flame atomic absorption spectrophotometry. N was measured using the Kjeldahl method.

### Determination of total volatile oil and volatile components

The content of volatile oil was determined according to the methods described in the general rule 2204 of the Chinese Pharmacopoeia (Volume IV), 2020 edition^34^. Volatile components were identified using the following steps: weigh 0.3 g of fresh *A. argyi* leaves; grind the leaves; place the product into a 20 mL headspace bottle of a solid-phase microextraction instrument; insert the manual sampler of pdms-dvb extraction fiber head (Supelco, USA) into the bottle; balance at 85℃ for 10 min; push out the extraction head; empty the extract for 40 min; remove the extract and immediately insert it into the sample inlet of the chromatograph; and desorb for 3 min. The conditions of the gas chromatography-mass spectrometer (GC–MS, Thermo Scientific, USA) were determined based on a previous study by Chen et al.^15^. The Mainlib library was used to search for each chromatographic peak in the total ion flow diagram of volatile oil by GC–MS. The chromatographic peaks with a probability > 50% were selected for further analysis.

### Determination of total phenolic acids and flavonoids and their components

The total phenolic acid and flavonoid content in fresh *A. argyi* leaves were determined according to Estévez et al.^35^. Extracts from each sample were collected through the ultrasonication of 0.2 g (passed through a 40-mesh sieve) of leaf tissue combined with 50 mL of 60% methanol for 40 min. The resulting extract was then filtered and utilized for the quantification of total phenols and total flavonoids. To determine the total flavonoids, 2 mL of the sample extract was transferred into a 25 mL flask and combined with 5 mL of distilled water and 1 mL of 5% NaNO_2_ solution. The contents were shaken for 6 min to ensure a thorough mixture. Next, 1 mL of 10% Al(NO_3_)_3_ solution was added and sitting for an additional 6 min. The solution was then mixed with 10 mL of 4% NaOH and the final volume was adjusted to the 25 mL mark by adding distilled water. The sample was allowed to stand at room temperature for 45 min before being measured for absorbance at 509 nm using rutin as the standard. Total phenols contents were measured by adding 1 mL of the sample extract into a 25 mL brown flask with 11 mL of distilled water, followed by 1 mL of Florinol reagent. The contents were mixed for 1 min and 20% NaCO_3_ solution was added until the mark 25 mL mark was reached. The mixture was shaken well and allowed to stand for 50 min before being measured for absorbance at 750 nm using gallic acid as the standard. The Agilent 1260 Infinity HPLC system (Agilent Technologies) was used for all HPLC analyses. The phenolic acids and flavonoids were separated using the ZORBAXRRHD Eclipse Plus 95A C18 column (2.1 9 100 mm, 1.8 lm, Agilent) and detected at 330 nm by UV. The mobile phases were acetonitrile (A) and 0.1% phosphoric acid (B) with a flow rate of 0.4 mL/min. The separation gradient conditions were as follows: 0–0.5 min, 2–5% A; 0.5–7 min, 5–25% A; 711 min, 25–33% A; 11–14 min, 30–33% A; 14–19.5 min, 33– 45% A; 19.5–20.5 min, 45–85% A; 20.5–27 min, 85–98% A; 27–28 min, and 98–2% A^36^.

### Total RNA extraction and RNA-seq analysis

The total RNA of leaves from each *A. argyi* sample was isolated using a Trizol reagent (Invitrogen, Carlsbad, CA, USA) with three biological replicates. cDNA libraries were constructed using 5 μg of RNA per sample and sequenced using the Novaseq 6000 platform. After data filtering, clean reads were mapped to the reference genome using the HISAT 2.2.4 and the Bowtie2 programs. The reference genome can be found at https://iris.angers.inra.fr/gddh13/index.html. The fragment per kilobase transcript per million mapped reads (FPKM) values for each transcribed area were calculated using the RESM program. A fold change > 2 and a divergence probability < 0.8 were used to identify differentially expressed genes (DEGs) with the DESeq2 software. The screening criteria of differentially expressed genes were |log_2_FC|≥ 1.00 using an adjusted p-value ≤ 0.05. Enrichment analysis was performed using annotations from the Kyoto Encyclopedia of Genes and Genomes (KEGG) and Gene Ontology (GO).

### RT-qPCR analysis

*A. argyi* genes involved in photosynthesis and the synthesis of phenolic acids, flavonoids, and terpenes in leaves were selected for quantitative real-time reverse transcriptase PCR (RT-qPCR) detection. The primers used for this analysis are listed in Table S1. Total RNA was reverse transcribed to cDNA using the ACTIN gene (5′-CGACTATGTTCCCTGGTATTGC-3′ and 5′-AGACCCTCCGATCCAGACACT-3′) as the internal reference. The primers were designed using the NCBI primer designing tool (https://www.ncbi.nlm.nih.gov/tools/primer-blast/index.cgi?LINK_LOC=BlastHome). The RT-qPCR was performed using a LightCycler 480 Instrument II and a tiangen real universal Color Premix (SYBR green). The 2^ΔΔCT^ method was used to calculate the relative expressions of each gene. A total of three biological replicates were used for each sample.

### Statistical analysis

Data processing and mapping were performed using GraphPad Prism 9.00 software. All data were presented as mean ± standard deviation (SD). Each treatment was represented by at least three biological replicates. One-way ANOVA and LSD (Least Significant Difference) tests were carried out using the SPSS 20.0 statistical software. Regression models were established to estimate curvatures and Pearson’s correlation coefficient, was conducted using SPSS 20.0 software.

### Supplementary Information

Supplementary Information.

## Data Availability

The datasets presented in this study can be found in online repositories https://www.ncbi.nlm.nih.gov/genome/. The raw RNA-seq data has been deposited into NCBI under the accession number PRJNA932574.
