# Supplementary material for: Mechanisms governing the impact of nitrogen stress on the formation of secondary metabolites in Artemisia argyi leaves
Source: Sci Rep. 2023 Aug 8;13:12866. doi: 10.1038/s41598-023-40098-5 (PMC10409738; doi:10.1038/s41598-023-40098-5)
Supplement: Supplementary file 1 — Supplementary Information. [file 41598_2023_40098_MOESM1_ESM.docx]

**Table S1**. The forward primer and reverse primer of different genes to RT-qPCR

| No. | Gene-number | Forward primer | Reverse primer |
| --- | --- | --- | --- |
| 1 | AY148678-RA | AATGCATGCGGTTTCTCCAC | TAAGCACGTGTCTACTCGCC |
| 2 | AY067952-RA | ACTGGTCAATGCGTGGTTCT | CCGACGCCAAAGGGTAAGTA |
| 3 | AY177451-RA | CAACCTCGCTTGATGGTTCAC | CTGGAGAAACACCATGCTTCC |
| 4 | AY028393-RA | TTTACCCGGGGGAGAAAAGC | CTAGGCTCATACCCGCACAT |
| 5 | AY009396-RA | TAAGCTCGGTCTCAAGGAGGA | AGTCAAACCAGGACCGAACC |
| 6 | AY263333-RA | CCTGACTCGGAAGGAGCAAT | CCAGAATAGCTGGACCACCA |
| 7 | AY028715-RA | CTGGTGGTAAGCGAGTGAGG | TGACCCGCTACCAAACCTTC |
| 8 | AY150191-RA | AAACCTCGCTCGTCTTTCGT | CAACAATCCTGAACCACGGC |
| 9 | AY178405-RA | ACTAGAGCGCCGGTTGTTAG | GCGTTTGAGCCTGGTGATTC |
| 10 | AY078091-RA | TCAATTGGAGGGTTCGGCTC | CCTCCAAAGCCTCTCGTGTT |
| 11 | AY065247-RA | GGTGAGGTTGTTGACCCACT | GGTCCCTTTCCGGTCACAAT |
| 12 | AY241096-RA | CGTCAACAGGACCACCTCTG | GGTGCTCACGGTAGACGAAA |
| 13 | AY070796-RA | ATCTACTTCGCCTCATCGCC | ATCATCACACGCCTCTGCAA |
| 14 | AY163355-RA | TCGGTACTCTTTTGGGCACC | GTCATCCCGTTCTGCTCACA |
| 15 | AY175527-RA | ATCCGTGGGTGACAAGCTAC | TGGCCTTCGTAAAATCCCCA |
| 16 | AY241097-RA | CAACATCGGCTTCCCAGTCA | AAGGAACCCACTTGTTGCGA |
